# Scar/WAVE has Rac GTPase-independent functions during cell wound repair

**DOI:** 10.1038/s41598-023-31973-2

**Published:** 2023-03-23

**Authors:** Mitsutoshi Nakamura, Justin Hui, Viktor Stjepić, Susan M. Parkhurst

**Affiliations:** Basic Sciences Division, Fred Hutchinson Cancer Center, 1100 Fairview Ave N, Seattle, WA 98109 USA

**Keywords:** Cell biology, Cytoskeleton, Actin

## Abstract

Rho family GTPases regulate both linear and branched actin dynamics by activating downstream effectors to facilitate the assembly and function of complex cellular structures such as lamellipodia and contractile actomyosin rings. Wiskott-Aldrich Syndrome (WAS) family proteins are downstream effectors of Rho family GTPases that usually function in a one-to-one correspondence to regulate branched actin nucleation. In particular, the WAS protein Scar/WAVE has been shown to exhibit one-to-one correspondence with Rac GTPase. Here we show that Rac and SCAR are recruited to cell wounds in the *Drosophila* repair model and are required for the proper formation and maintenance of the dynamic actomyosin ring formed at the wound periphery. Interestingly, we find that SCAR is recruited to wounds earlier than Rac and is still recruited to the wound periphery in the presence of a potent Rac inhibitor. We also show that while Rac is important for actin recruitment to the actomyosin ring, SCAR serves to organize the actomyosin ring and facilitate its anchoring to the overlying plasma membrane. These differing spatiotemporal recruitment patterns and wound repair phenotypes highlight the Rac-independent functions of SCAR and provide an exciting new context in which to investigate these newly uncovered SCAR functions.

## Introduction

Cells encounter physical stresses daily leading to breaks in their cortex that must be rapidly repaired to maintain cell integrity and function^1–4^. One of the major features of cell wound repair is the assembly of a Rho family GTPase-dependent actomyosin ring at the wound periphery that attaches the cortical cytoskeleton to the overlying plasma membrane, followed by its dynamic translocation inward to pull the cell cortex breach closed^5–10^.

The three major Rho family GTPases—Rho, Rac, and Cdc42—are recruited to wounds in spatially and temporally distinct patterns to carry out specific functions during the repair process^7–9,11^. This dynamic recruitment of Rho family GTPases to the wound site varies somewhat among cell wound repair model systems. In the *Xenopus* model, all three GTPases are immediately recruited to the wound where they form two concentric rings with an interior ring consisting of Rho and an outer ring containing both Cdc42 and Rac^8,9,12^. In the *Drosophila* model, Rho GTPases are also rapidly recruited to wounds in concentric rings, but they exhibit both spatial and temporal differences^7,13^. Rho1 accumulates first at 30 s post-wounding, and similar to that observed in the *Xenopus* model, becomes enriched in a ring inside of and just overlapping with the inner edge of the actin ring. Cdc42 accumulates next (30–60 s post-wounding), whereas Rac 1 and Rac 2 accumulate last (60–90 s post-wounding). While both Cdc42 and Rac recruitment overlap with the actin ring, Rac also shows an additional broad region of slightly elevated accumulation overlapping the actin halo region.

While all three Rho family GTPases are needed to achieve proper cell wound repair, their functions also vary somewhat between cell wound repair models. In the *Xenopus* model, RhoA is needed for cortical flow, whereas Cdc42 regulates actin ring formation, and both are involved in actin ring translocation^8,9,12^. Rac function in this model has not been reported. In the *Drosophila* model, Rac proteins are necessary for actin recruitment to the wound edge, Cdc42 is needed to stabilize the actin ring at the wound periphery, and Rho1 is involved in actomyosin ring assembly/stabilization^8,13^.

Rho family GTPases are known to regulate actin dynamics through their regulation of linear and branched actin nucleation factors^14–19^. One such family of actin nucleation factors, the Wiskott-Aldrich Syndrome (WAS) family (WASp, Scar/WAVE, WASH), works with the Arp2/3 complex to promote branched actin nucleation^15,19–25^. In the context of cell wound repair, WAS family proteins have been shown to contribute to actin filament orientation and nucleate branched actin to serve as a scaffold to assemble and maintain the contractile actomyosin cable at the wound periphery^10,19^.

As Rho family GTPases usually exhibit a one-to-one correspondence with WAS family proteins (Cdc42 > WASp; Rac > Scar/WAVE; Rho1 > Wash)^22,26–36^, we expected Rac and SCAR to show similar recruitment to wounds and repair phenotypes. Surprisingly, we find that SCAR is recruited to wounds prior to Rac, does not require Rac for its recruitment to wounds, and exhibits Rac-independent repair phenotypes, suggesting that Rac and SCAR are functioning outside of their usual relationship in the cell wound repair context.

## Results

### SCAR is recruited to cell wounds before Rac GTPase

**Figure 1:**
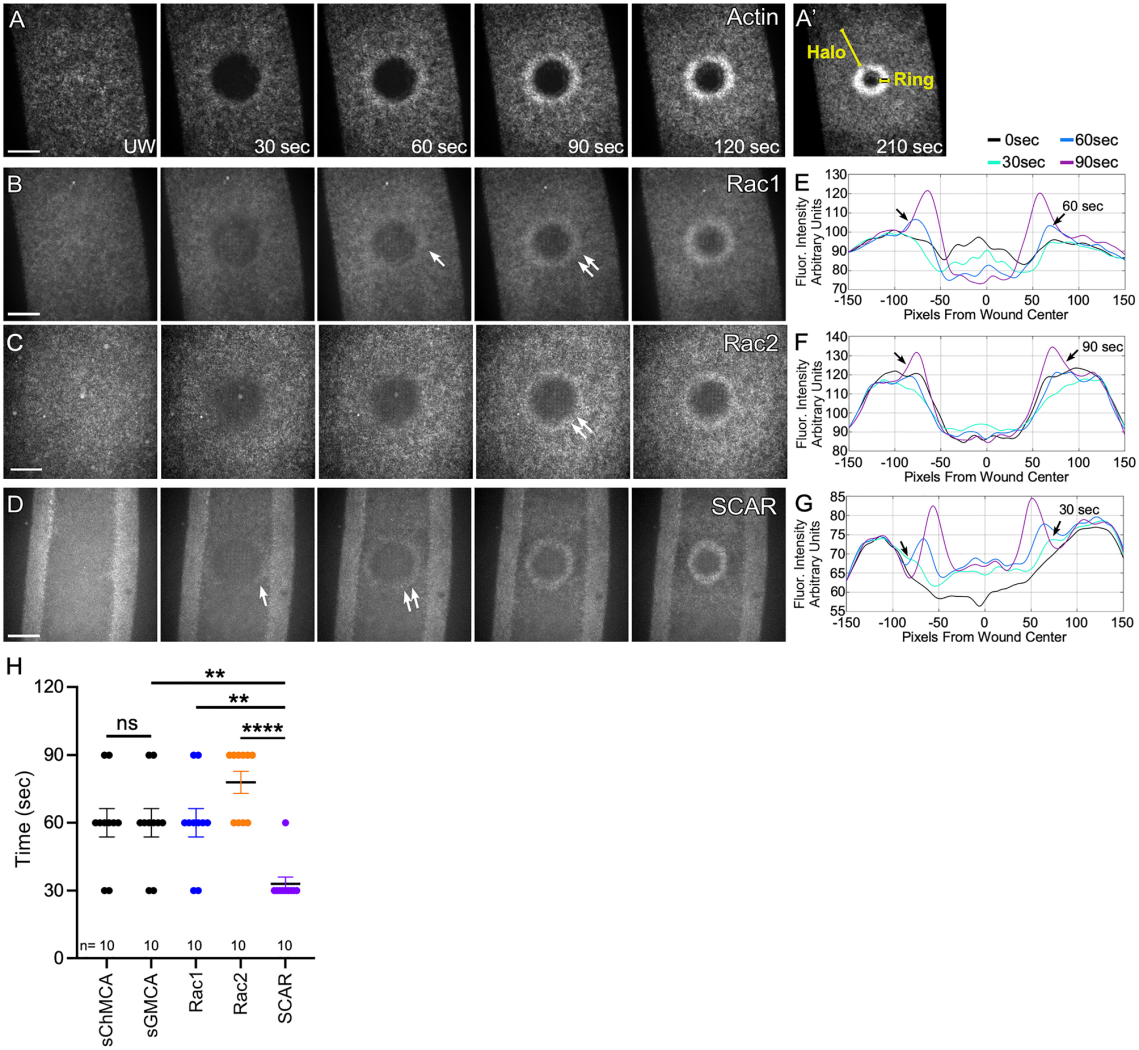
SCAR is recruited to cell wounds faster than Rac1 or Rac2. Confocal projection images of embryos expressing an actin reporter (sGMCA; **A**-**A’**) or co-expressing mCherry-Rac1 and sGMCA (**B**), GFP-Rac2 and sStMCA (**C**), and GFP-SCAR and sStMCA (**D**). Times post-wounding are indicated. UW = unwounded. Arrows indicate recruitment to the wound periphery. (**E**–**G**) Fluorescence intensity (arbitrary units) profiles across the wound area over time for the images shown in (**B**–**D**), respectively. (**H**) Dotplot of initial actin, Rac1, Rac2, and SCAR recruitment to wounds. Scale bars: 20 μm.

In the Drosophila cell wound repair model, actin is recruited to cell wounds in a dense ring around the wound periphery and in a less dense actin halo at 60 ± 6.3 s post-wounding (Fig. 1A-A’,H)^37^. Wounds were generated by laser ablation on the lateral side of nuclear cycle 4–6 *Drosophila* syncytial embryos expressing an actin reporter (sGMCA or sChMCA;^38^) (see “Methods”). Consistent with its requirement for cell wound repair, Rac is recruited to the wound edge. Wounding embryos expressing fluorescently tagged Rac1 (ChFP-Rac1) or Rac2 (GFP-Rac2) under the control of their endogenous promoters results in the strong accumulation of these proteins beginning at 60 ± 6.3 and 78 ± 4.9 s post-wounding, respectively, in a ring encircling the wound and in a less intense concentric ring corresponding to the actin ring and halo regions (Fig. 1B,C,E,F,H; Table 1)^7,13^.

**Table 1:** Time when each reporter is first recruited to the wound periphery.

Genotype	Mean (s)	SEM	p-value to SCAR	p-value to sGMCA
sChMCA	60	6.3	–	ns
sGMCA	60	6.3	< 0.01	–
Rac1	60	6.3	< 0.01	–
Rac2	78	4.9	< 0.0001	–
SCAR	33	3	–	–

Statistical tests were performed using Kruskal–Wallis test.

As Rho family GTPases usually exhibit a one-to-one correspondence with WAS family proteins (Rac1 > Scar/WAVE), we expected SCAR to show similar spatial and slightly delayed temporal recruitment to wounds as Rac. Wounding embryos expressing GFP-tagged SCAR (GFP-SCAR) under the control of its endogenous promoter resulted in the recruitment of SCAR to the wound periphery (Fig. 1D,G,H; Table 1). Surprisingly, SCAR is recruited to wounds earlier (33 ± 3 s post-wounding) than that observed for Rac1/Rac2 (60 s and 78 s post-wounding, respectively) (Fig. 1E,G,H; Table 1) and only overlapping the intense actin ring encircling the wound (i.e., absent from the actin halo region). Thus surprisingly, Rac1/Rac2 and SCAR recruitment to wounds is both temporally distinct and only partially overlapping spatially, suggesting that SCAR has Rac-independent functions in the context of cell wound repair.

### SCAR recruitment to cell wounds does not require Rac activity

**Figure 2:**
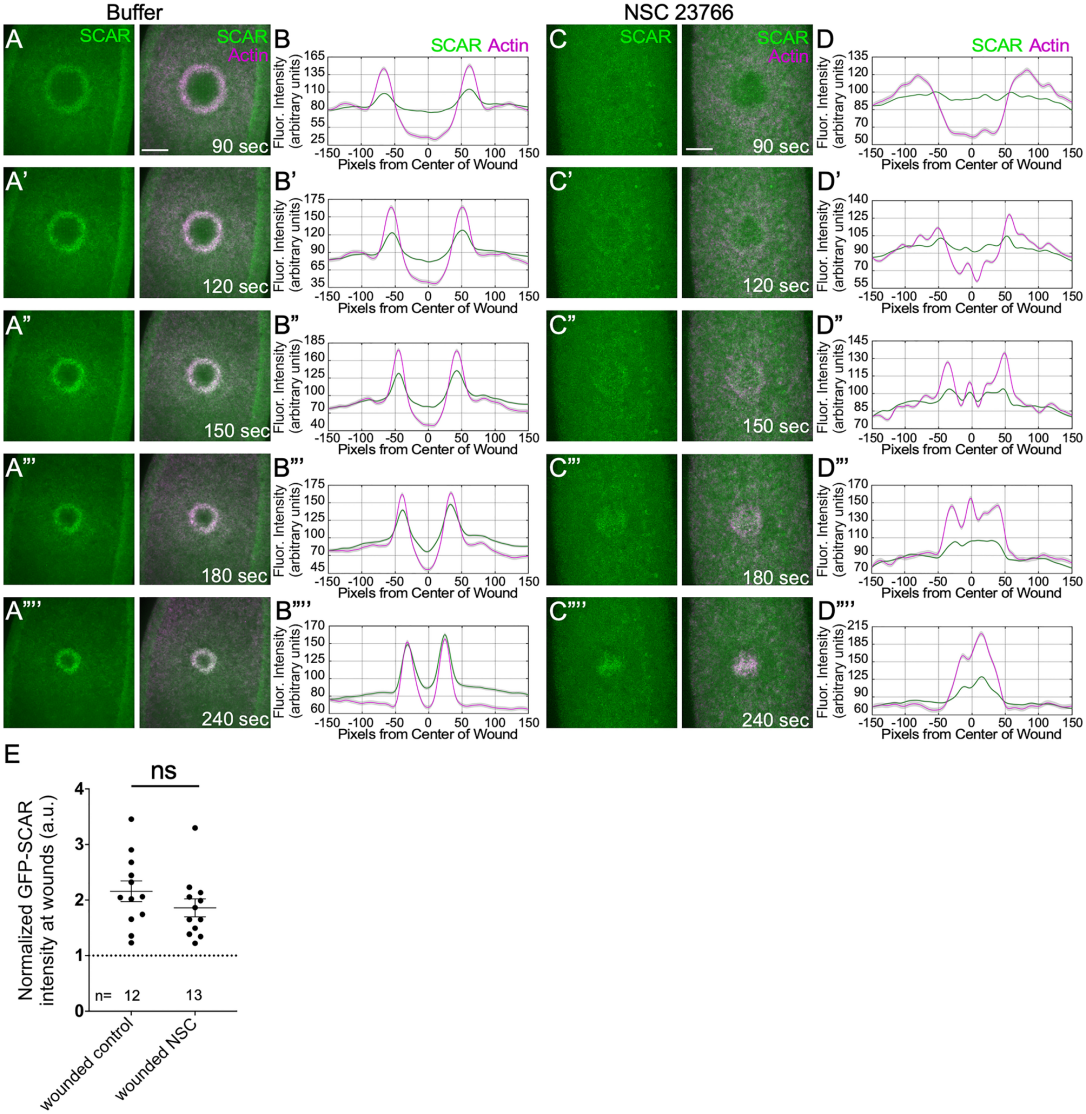
SCAR is recruited to cell wounds in the absence of Rac activity. (**A**-**A””**) Confocal projection images of buffer-injected embryos co-expressing GFP-SCAR and an actin reporter (sStMCA) at the time points indicated. (**B**-**B””**) Fluorescence intensity (arbitrary units) profiles across the wound area in (**A**-**A””**), respectively. (**C**–**C””**) Confocal projection images of NSC 23766-injected embryos co-expressing GFP-SCAR and an actin reporter (sStMCA) at the time points indicated. (**D**-**D””**) Fluorescence intensity (arbitrary units) profiles across the wound area in (**C**–**C””**), respectively. (**E**) Dotplot of GFP-SCAR recruitment to wounds in control (buffer-injected) and NSC 23766-injected embryos at 240 s post-wounding. Scale bars: 20 μm.

To further investigate the relationship between Rac and SCAR, we asked if recruitment of SCAR to cell wounds requires Rac. As *Drosophila* has three Rac genes (Rac1, Rac2, and Mtl), we modulated Rac activity by treating embryos with the potent Rac inhibitor NSC 23766^7,39^. We examined GFP-SCAR recruitment to wounds in embryos injected with NSC 23766 (Fig. 2). Consistent with their different temporal recruitment patterns, GFP-SCAR was still recruited to wounds in these Rac inhibited embryos. The GFP-SCAR that is recruited to cell wounds does not form a well-defined actin ring, likely due to reduced actin recruitment to wounds upon Rac inhibition. Our results indicate that SCAR function is Rac-independent during cell wound repair.

### Knockdown of Rac or SCAR results in distinct defects in wound healing dynamics

All three Rho family GTPases are required non-redundantly for cell wound repair. In particular, Rac is needed for the recruitment of actin to the wound edge, while Rho1 and Cdc42 are crucial for the formation and stability of the actin ring^7–9,13^. Interestingly, some actin organization persisted at the wound edge in embryos where Rho1 or Diaphanous (Dia; *Drosophila* formin protein and Rho1 downstream effector) were knocked down^7^, consistent with the differing roles for Rac and/or Cdc42 in the repair process.

**Figure 3:**
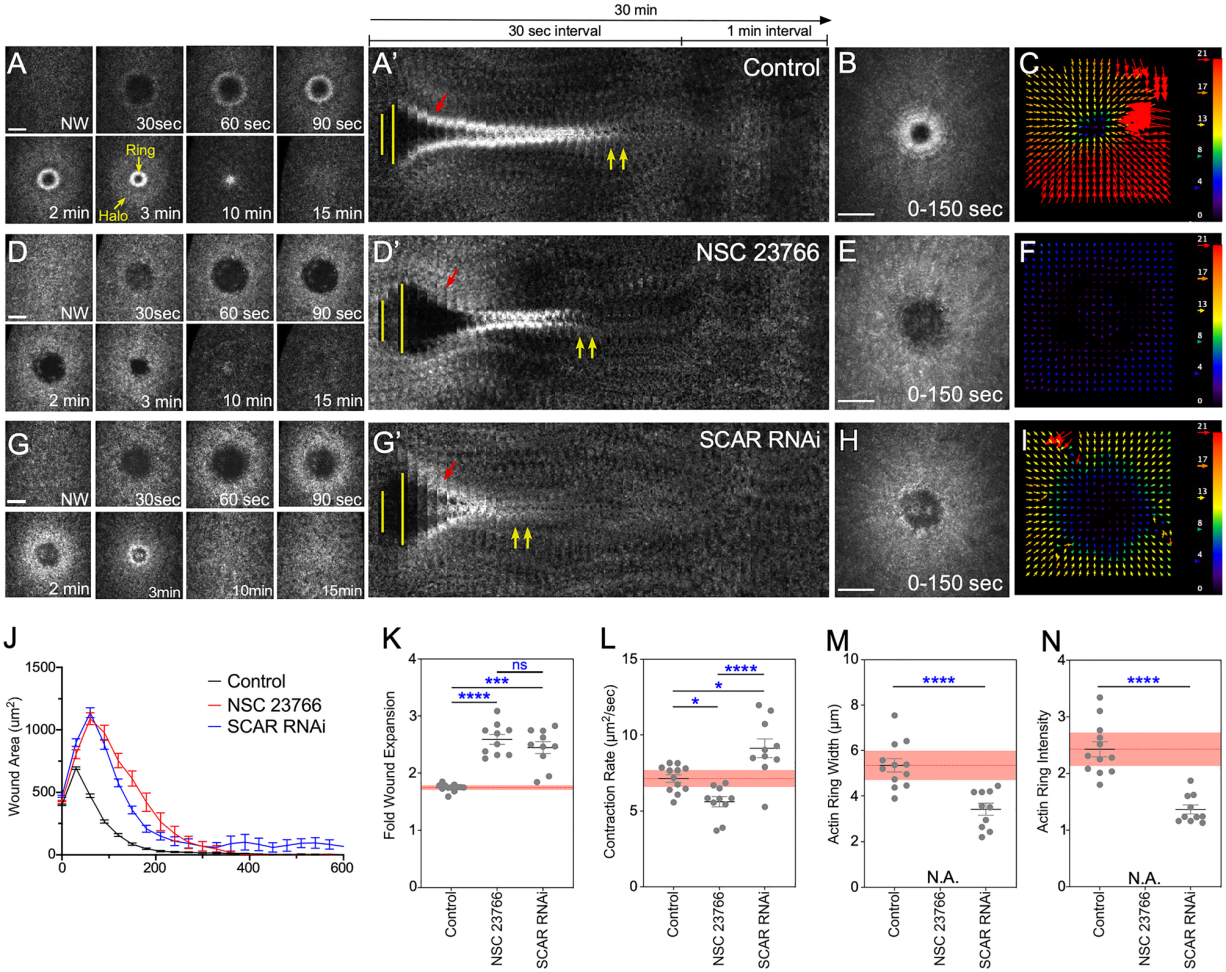
Rac and SCAR knockdowns exhibit different cell wound repair phenotypes. (**A**–**I**) Confocal projection images of wounds generated in embryos expressing an actin marker (sGMCA) in control (buffer only; **A**–**C**), NSC 23766 injected (potent Rac inhibitor; **D**–**F**), or SCAR RNAi knockdowns (**G**–**I**). Actin ring and halo are indicated in (**A**). (**A’**,**D’**,**G’**) Kymographs across the wound area in (**A**,**D**,**G**), respectively. Wound expansion is noted with yellow lines; actomyosin ring formation with red arrows; and actomyosin ring disassembly with yellow arrows. (**B**,**E**,**H**) XY projection image at 0–150 s post wounding showing cortical flow of actin to the wound edge. (**C**,**F**,**I**) Vector maps from PIV (Particle Image Velocimetry) analysis depicting actin flow from 60 to 90 s for (**A**,**B**,**D**,**E**,**G**,**H**) respectively. (**J**) Quantification of the wound area over time for control (buffer injected), NSC 23766 injected, and SCAR RNAi knockdowns. (**K**–**N**) Quantification of fold wound expansion (**K**), wound contraction rate (**L**), actin ring width (**M**), and actin ring intensity (**N**). Black line and error bars represent mean ± SEM. Red dotted line and square represent mean ± 95% CI from control. Kruskal–Wallis test (**K**–**L**) and Welch’s t-test (**M**–**N**) were performed with * is p < 0.05, ** is p < 0.01, *** is p < 0.001, **** is p < 0.0001, and ns is not significant. Scale bars: 20 μm.

We compared actin dynamics between embryos injected with NSC 23766 and SCAR knockdowns (Fig. 3; Table 2). In control (buffer injected) embryos, actin accumulates similarly to wildtype: in a highly enriched actomyosin ring bordering the wound edge and an elevated actin halo encircling the actin ring (Fig. 3A-C, J-L; Table 3; Video 1). As shown previously, Rac inhibition results in severely reduced recruitment of actin to the wound edge such that an actin ring is not formed, and significantly slower wound closure (control: 7.15 ± 0.25 μm^2^/s; NSC 23766: 5.62 ± 0.34 μm^2^/s, p < 0.05) (Fig. 3D–F,J–N; Table 3; Video 1)^7^. Rac inhibition also results in overexpansion of wounds (control: 1.74 ± 0.02 fold; NSC 23766: 2.59 ± 0.09 fold, p < 0.0001) (Fig. 3D-D’,K; Table 3). These phenotypes would be consistent with a role for branched actin nucleation factors as downstream effectors of Rac (and Cdc42) in this process.

**Table 2:** Knockdown efficiency of the SCAR RNAi line used in this study.

Sample	ATPα Signal	SCAR Signal	Normalized SCAR Signal	% Knockdown
Control* (rep1)	34,991.86	30,755.07	–	–
SCAR RNAi (rep1)	15,017.69	1914.48	4460.81	86%
Control* (rep 2)	39,264.17	42,224.32	–	–
SCAR RNAi (rep 2)	23,228.42	4102.68	6934.97	84%

*Control refers to *vermillion* (unrelated gene) knockdown.

**Table 3:** Actin ring dynamics in the three genotypes shown in Fig. 3.

	Control*			NSC 23766				SCAR RNAi			
	Mean	SEM	p-value to control	Mean	SEM	p-value to control	p-value to SCAR RNAi	Mean	SEM	p-value to control	p-value to NSC 23766
Wound Fold Expansion	1.74	0.018	–	2.59	0.09	< 0.0001	ns	2.45	0.11	< 0.001	ns
Contraction Rate (μm^2^/sec)	7.15	0.25	–	5.62	0.34	< 0.05	< 0.0001	9.12	0.62	< 0.05	< 0.0001
Actin Ring Width (μm)	5.34	0.29	–	–	–	–	–	3.43	0.26	< 0.0001	–
Actin Ring Intensity	2.43	0.13	–	–	–	–	–	1.34	0.08	< 0.0001	–

*Control refers to *vermillion* (unrelated gene) knockdown. Statistical tests were performed using Kruskal–Wallis test.

SCAR has been shown to contribute to actin filament orientation during actomyosin ring formation and translocation^40^, which would necessarily occur after actin recruitment to the wound periphery. Consistent with this and its Rac-independent recruitment to wounds, SCAR knockdowns exhibit different wound repair dynamics phenotypes from that of Rac inhibition. SCAR knockdowns also result in wound over-expansion (2.45 ± 0.11 fold, p < 0.001), but they exhibit a faster wound closure rate (9.12 ± 0.62 μm^2^/sec) than both control (p < 0.05) and Rac inhibited (p < 0.0001) embryos (Fig. 3G-G’, J-L; Table 3; Video 1). Importantly, unlike Rac inhibition, actin is recruited to the wound edge in SCAR knockdowns (Fig. 3H–I; Table 3). Despite this actin recruitment, the actomyosin ring that is formed is not as tightly organized as that in wildtype (Fig. 3G-H,M–N; Table 3), and the actomyosin ring undergoes premature disassembly (Fig. 3G’; Table 3).

## Discussion

While Rac and SCAR are both needed for proper cell wound repair, they are doing so outside of their “Rac is the canonical activator of SCAR” relationship in this context. We find that Rac is necessary for actin recruitment to cell wounds, whereas SCAR is recruited to cell wounds in the absence of Rac activity and affects actin ring organization and contraction.

Scar/WAVE forms a heteropentameric complex with the Wave Regulatory Complex (WRC) composed of Sra1/PIR121, Nap1, Abi, and HSPC300^25,28,41^. Scar/WAVE, along with the Arp2/3 complex, can generate branched actin networks^26,27,42,43^. The Scar/WAVE–WRC complex is thought to be trans-inhibited^44–46^. Rac binds to the WRC to relieve this trans-inhibition, allowing Scar/WAVE to work with Arp2/3 to nucleate branched actin^27–29,44^. Rac does not regulate Scar/WAVE by binding directly to it, but rather interacts with it through binding to the Sra1 subunit of its WRC^29^. Actin is recruited to the wound periphery in SCAR knockdowns, but does not assemble into a robust actomyosin ring. Thus, in the context of cell wound repair, SCAR could bypass the need for Rac by a currently unknown mechanism such that it can use its branched actin nucleation activity. In this scenario, SCAR may need to be present at the wound edge quickly to put a branched actin scaffolding in place, such that additional actin can be organized into a robust ring upon its arrival at the wound edge. Alternatively, SCAR could be using an, as yet undescribed, alternate biochemical activity when at the wound edge. For example, in addition to its branched actin nucleation activity, the related Wiskott-Aldrich Syndrome family member WASH has been shown to bundle actin, bundle microtubules, and crosslink them^35^.

Surprisingly, despite the disruption of the actin ring, wounds in SCAR knockdowns close faster than in controls. A recent study showed that actomyosin contraction force is dependent on F-actin architectures^47^. While branched actin nucleation stabilizes F-actin networks by increasing their density and connectivity, this stabilization reduces actomyosin contractility by limiting movement of the myosin motor on F-actin. The three major branched actin nucleators (WASp, SCAR, and WASH) are required to form a robust actomyosin ring during cell wound repair in the *Drosophila* model^19^. While the actomyosin ring is disrupted in SCAR knockdowns, WASp and WASH could still generate branched F-actin to support the formation of an actomyosin ring at the wound edge. This disrupted actomyosin ring could result in increased actomyosin contractility due to its reduced F-actin network density and connectivity, thereby leading to the faster wound closure in SCAR knockdowns.

While Rac has known effectors other than Scar/WAVE that could work downstream to mediate its role in actin recruitment to wounds (cf.^17,48,49^), less is known about Scar/WAVE regulation outside of its activation through Rac GTPases. Strikingly, our data suggest that SCAR localization and its activity are not always dependent on Rac GTPase, which raises new mechanistic questions for SCAR/WAVE function. What protein(s) regulate SCAR recruitment to the cell cortex without Rac GTPase? While active Rac GTPase regulates SCAR activity through binding to Sra1 (WRC subunit), what other proteins can change the conformation of SCAR to release its VCA domain to promote branched actin nucleation? The linear actin nucleation factor Diaphanous (Dia) has been shown to function upstream of SCAR to regulate WRC localization and activity during *Drosophila* myoblast fusion^50,51^. Consistent with this possible regulation, Dia is rapidly recruited to the wound edge at 30 s post wounding, similar to that of SCAR^40^. SCAR may also depend on another Rho family GTPase. Cip4, a Cdc42 downstream effector, has been shown to function upstream of SCAR via associating with the WRC to control Dynamin-dependent cell polarization in the *Drosophila* wing^52^. Interestingly, recent studies have also implicated other means of activating the WRC in specific contexts such as lamellipodia formation through interaction with factors including Arf-family GTPases (Arf1, Arf6), other Rho family GTPases (Cdc42, RhoG), various kinases, phospholipids, or membrane receptors^25,31,53–57^. The identification of these instances of Rac-independent regulation for Scar/WAVE provides exciting new entry points for investigating the upstream control of this essential branched actin nucleation promoting factor.

## Materials and methods

### Fly stocks and genetics

Flies were cultured and crossed at 25 °C on yeast-cornmeal-molasses-malt extract medium. Flies used in this study are: ChFP-Rac1 (BDSC #76266)^13^, GFP-Rac2 (BDSC #52286)^7^; SCAR-GFP^40^, Vermillion RNAi (BDSC #50641; TRiP.HMC03041), and SCAR RNAi (BDSC #51803; TRiP.HMC0336). RNAi lines were driven using the maternally expressed GAL4-UAS driver, Pmatalpha-GAL-VP16V37 (BDSC #7063).

An actin reporter, sGMCA (spaghetti squash driven, moesin-alpha-helical-coiled and actin binding site fused to GFP) reporter^38^, the mCherry fluorescent equivalent, sChMCA (BDSC #35520), or the mScarlet-i fluorescent equivalent, sStMCA (BDSC #90928)^58^, was used to follow wound repair dynamics of the cortical cytoskeleton.

In this study, we used an actin reporter + maternal GAL4 driver + *vermilion* RNAi (unrelated fly RNAi) + injection buffer as the control.

Localization patterns and mutant analyses were performed at least twice with independent genetic crosses and ≥ 10 embryos were examined. Images representing the average phenotype were selected for figures.

### Western Blotting

To generate embryo whole cell lysates, 10 nuclear cycles 4–6 embryos were collected, dechorionated, and then homogenized in 2X sample buffer (125 mM Tris–Cl pH 6.8, 4% SDS, 0.1% Bromophenol blue, 20% glycerol). Western blotting was performed according to standard procedures using anti-SCAR (P1C1, 1:10)^59^ antibodies, with anti-ATP5A (15H4C4; 1:50,000; Abcam) for the loading control.

### Embryo handling and preparation

Nuclear cycle 4–6 embryos were collected for 30 min at 25 °C and harvested at room temperature (22 °C). Collected embryos were dechorionated by hand, mounted onto No. 1.5 coverslips coated with glue, and covered with Series 700 halocarbon oil (Halocarbon Products Corp.) as previously described^37^.

### Drug injections

The pan Rac GTPase inhibitor NSC 23766 (50 mM; Tocris Bioscience) was injected into NC4-6 staged *Drosophila* embryos, incubated at room temperature (22 °C) for 5 min, and then subjected to laser wounding. NSC23766 was prepared in injection buffer (5 mM KCl, 0.1 mM NaP pH6.8). Injection buffer alone was used as the control.

### Laser wounding

All wounds were generated using a pulsed nitrogen N2 micropoint laster (Andor Technology Ltd.) set to 435 nm and focused on the lateral surface of the embryo. An 18 × 18 μm circular region was set as the target site along the lateral midsection of the embryo, and ablation was controlled by MetaMorph software (Molecular Devices). Average ablation time was less than 3 s and time-lapse image acquisition was initiated immediately after ablation.

### Live image acquisition

All live imaging was performed at room temperature with the following microscope:

1. Revolution WD systems (Andor Technology Ltd.) mounted on a Leica DMi8 (Leica Microsystems Inc.) with a 63x/1.4 NA objective lens under the control of MetaMorph software (Molecular devices). Images were captured using 488 nm and/or 561 nm lasers with a Yokogawa CSU-W1 confocal spinning disk head attached to an Andor iXon Ultra 897 EMCCD camera (Andor Technology Ltd.). Time-lapse images were acquired with 17–20 µm stacks/0.25 µm steps. Images were acquired every 30 s for 15 min and then every 60 s for 15 min.
2. UltraVIEW VoX Confocal Imaging System (Perkin Elmer, Waltham, MA, USA) mounted on a Nikon Eclipse Ti (Nikon Instruments, Melville NY,USA) with a 60x/1.4 NA objective lens under the control of Volocity software(v.5.3.0, Perkin-Elmer). Images were captured using 488 nm and/or 561 nm lasers with a Yokogawa CSU-X1 confocal spinning disk head attached to a Hamamatsu C9100-13 EMCCD camera (Perkin-Elmer, Waltham,MA,USA). Time-lapse images were acquired with 17–20 µm stacks/0.25 µm steps. Images were acquired every 30 s for 15 min and then every 60 s for 15 min.

### Image processing, analysis, and quantification

Image processing was performed using FIJI software^60^. In all images, the top side is anterior and the bottom side is posterior of embryos. Kymographs were generated using the crop feature to select ROIs of 5.3 × 94.9 µm. Wound area was manually measured using Fiji and the values were imported into Prism 8.2.1 (GraphPad Software Inc.) to construct corresponding graphs.

For fluorescent lineplots, the mean fluorescence profile intensities were calculated from 51 equally spaced radial profiles anchored at the center of the wound, swept from 0° to 180°^40^. Radial profiles of 301-pixel diameter were used. Fluorescence intensity profiles were calculated and averaged using an in house code using MATLAB R2020b (MathWorks) (available at: https://github.com/FredHutch/wound_radial_lineplot), then plotted using MATLAB R2020b. For dynamic lineplots, we generated fluorescent profile plots from each timepoint and then concatenated them. The lines represent the averaged fluorescent intensity and gray area is the 95% confidence interval.

Quantification of the width and average intensity of actin ring, wound expansion, and closure rate was performed as follows: the width of actin ring was calculated with two measurements, the feret diameters of the outer and inner edge of actin ring at 90 s post-wounding. Using these measurements, the width of actin ring was calculated with (outer feret diameter − inner feret dimeter)/2. The average intensity of actin ring was calculated with two measurements. Instead of measuring feret diameters, we measured area and integrated intensity in same regions as described in ring width. Using these measurements, the average intensity in the actin ring was calculated with (outer integrated intensity − inner integrated intensity)/(outer area − inner area). To calculate relative intensity for unwounded (UW) time point, average intensity at UW was measured with 50 × 50 pixels at the center of embryos and then averaged intensity of actin ring at each timepoint was divided by average intensity of UW. Wound expansion was calculated with max wound area/initial wound size. Closure rate was calculated with two time points, one is t_max_ that is the time of reaching maximum wound area, the other is t < half that is the time of reaching 50–35% size of max wound since the slope of wound area curve changes after t < half. Using these time points, average speed was calculated with (wound area at t_max_ − wound area at t < half)/t_max_-t < half.

Figures were assembled in Canvas Draw 6 for Mac (Canvas GFX, Inc.).

### Statistical analysis

All statistical analysis was done using Prism 8.2.1 (GraphPad, San Diego, CA). Statistical significance was calculated using the Kruskal–Wallis or Welch’s t tests with * is p < 0.05, ** is p < 0.01, *** is p < 0.001, **** is p < 0.0001, and ns is not significant.

## Supplementary Information

Supplementary Video 1.

Supplementary Legends.

## Data Availability

All data generated and/or analyzed during this study are included in this published article and its supplementary video.
