# Supplementary material for: Scar/WAVE has Rac GTPase-independent functions during cell wound repair
Source: Sci Rep. 2023 Mar 23;13:4763. doi: 10.1038/s41598-023-31973-2 (PMC10036328; doi:10.1038/s41598-023-31973-2)
Supplement: Supplementary file 2 — Supplementary Legends. [file 41598_2023_31973_MOESM2_ESM.docx]

**Supplementary Video Legend**

**Video 1.** Rac and SCAR knockdowns exhibit distinct phenotypes in cell wound repair.

(A-C) Time-lapse confocal xy images and radial fluorescence intensity (arbitrary units) profiles across the wound area from *Drosophila* NC4-6 staged embryos expressing an actin marker (sGMCA): control (buffer injected; A), NSC 23766 injected (B), and SCAR RNAi (C). Time post-wounding is indicated. UW: unwounded
